# Synteny of *Prunus *and other model plant species

**DOI:** 10.1186/1471-2164-10-76

**Published:** 2009-02-10

**Authors:** Sook Jung, Derick Jiwan, Ilhyung Cho, Taein Lee, Albert Abbott, Bryon Sosinski, Dorrie Main

**Affiliations:** 1 Department of Horticulture and Landscape Architecture, Washington State University, Pullman, WA 99164, USA; 2 Computer Science Department, Saginaw Valley State University, University Center, MI 48710, USA; 3 Department of Genetics and Biochemistry, Clemson University, Clemson, SC 29634, USA; 4 Department of Horticultural Science, North Carolina State University, Raleigh, NC 27695, USA

## Abstract

**Background:**

Fragmentary conservation of synteny has been reported between map-anchored *Prunus *sequences and *Arabidopsis*. With the availability of genome sequence for fellow rosid I members *Populus *and *Medicago*, we analyzed the synteny between *Prunus *and the three model genomes. Eight *Prunus *BAC sequences and map-anchored *Prunus *sequences were used in the comparison.

**Results:**

We found a well conserved synteny across the *Prunus *species – peach, plum, and apricot – and *Populus *using a set of homologous *Prunus *BACs. Conversely, we could not detect any synteny with *Arabidopsis *in this region. Other peach BACs also showed extensive synteny with *Populus*. The syntenic regions detected were up to 477 kb in *Populus*. Two syntenic regions between *Arabidopsis *and these BACs were much shorter, around 10 kb. We also found syntenic regions that are conserved between the *Prunus *BACs and *Medicago*. The array of synteny corresponded with the proposed whole genome duplication events in *Populus *and *Medicago*. Using map-anchored *Prunus *sequences, we detected many syntenic blocks with several gene pairs between *Prunus *and *Populus *or *Arabidopsis*. We observed a more complex network of synteny between *Prunus*-*Arabidopsis*, indicative of multiple genome duplication and subsequence gene loss in *Arabidopsis*.

**Conclusion:**

Our result shows the striking microsynteny between the *Prunus *BACs and the genome of *Populus *and *Medicago*. In macrosynteny analysis, more distinct *Prunus *regions were syntenic to *Populus *than to *Arabidopsis*.

## Background

*Prunus *belongs to Rosaceae, the third most economically important plant family in the United States and other temperate regions of the world [1,2]. Within Rosaceae, *Prunus *contains the most diverse array of crops: fruits such as peach, apricot, plum, sweet cherry, and sour cherry, nuts like almond, and lumber trees like black cherry. Other important fruit producing crops in Rosaceae include apple (*Malus*), pear (*Pyrus*), raspberry/blackberry (*Rubus*) and strawberry (*Fragaria*). In addition, Rosaceae contains a wide variety of ornamental plants including roses, flowering cherry, crabapple and quince.

Significant conservation of the genomes among the *Prunus *member species has been shown by comparative mapping studies [3]. For example, comparisons of the anchor marker positions on the *Prunus *reference map with those on 13 other maps showed that the genomes of the diploid *Prunus *species are essentially collinear [3]. Large collinear blocks among different genera in Rosaceae, such as *Prunus *and *Malus*, were also detected [3]. Similar conserved collinearity of shared markers has also been observed among other closely related species within the grasses [4], legumes [5,6] and between potato and tomato in the Solanaceae [7].

With the increase in available genomic sequence data, more laboratories have looked at collinearity at the genome sequence level. The synteny between rice and other cereals are shown to be high [8], and substantial micro-collinearity among legumes was reported [9-11]. These comparisons have revealed that the conserved syntenic regions, detected by marker collinearity, were often interrupted by small genome rearrangements such as insertions, deletions, inversions and translocations [12-14]. Large scale sequence data has also enabled the detection of micro-collinear regions in less closely related species without apparent macrosynteny [15]. Small scale microsynteny was observed when a 276 kb region of the model legume *Medicago *(rosid I) was compared with the genomic sequence of *Arabidopsis *(rosid II) [16]. Conversely, no macrosynteny was found when the two genomes were compared using 172 mapped markers of *Medicago *[16]. Significant levels of local synteny were also detected covering segments of 1 Mb of *Arabidopsis *and regions of <5 cM in lettuce and sunflower, members of the asterids, even though the macrosyntenic patterns covering larger segments were not evident [17]. A recent study detected microsynteny between coffee, another asterid species, and the *Arabidopsis *genome using putative orthologous sequences and BAC ends [18]. We have previously detected conserved syntenic regions between *Prunus *and *Arabidopsis *using 475 peach ESTs anchored to *Prunus *genetic maps and 1097 peach ESTs anchored to BAC contigs [17]. The conserved syntenic regions were short and fragmentary, and often the *Prunus *regions matched to more than one *Arabidopsis *block. This complex network of microsynteny, often including non-collinear regions, between *Arabidopsis *and the distantly related species suggests multiple genome duplications followed by differential gene loss [15-21]. Large segmental or whole genome duplication followed by gene loss seems to be prevalent in the evolution of all flowering plant genomes, and the degree of genome instability seems to be higher in some species of Brassicaceae, such as *Arabidopsis*, and in some species of Poaceae as well [22-24].

Synteny analysis across species provides insight on the evolutionary relationships between different lineages and also the opportunity to study the relationship between genome structure and function of organisms. The micro-collinear regions conserved between model organisms like *Arabidopsis *and lesser-studied organisms can also facilitate marker saturation and candidate gene searches. For example, micro-collinearity data between *Arabidopsis *and rice has been utilized in the improvement of phylogenetic resolution of the expansin gene family [25]. Multiple rounds of polyploidization or large segmental duplication followed by gene loss, however, can greatly obscure the synteny, impeding the transfer of genomic knowledge from model species genomes to those of less well characterized species.

The recent availability of the whole genome sequence of *Populus trichocarpa *provides another resource in the detection of conserved synteny among plant genomes [26]. Also available are the partially sequenced *Medicago *genome [11]. *Populus *and *Medicago *belong to rosid I which also includes *Prunus*; hence *Prunus *is evolutionarily closer to them than *Arabidopsis *which belongs to rosid II. *Populus *is of particular interest to compare with *Prunus *since *Populus *is completely sequenced, is a fellow tree species and it appears to be more stable compared to the *Arabidopsis *genome; nucleotide substitution, tandem gene duplication and gross chromosomal rearrangement proceed more slowly in *Populus *then in *Arabidopsis *[26]. The detection of melon BAC regions that are more conserved in *Populus *than in *Medicago *or *Arabidopsis *[27] and the report of papaya BACs that are more collinear in *Populus *than in *Arabidopsis *[28] also suggest that the *Populus *genome may be useful in the exploration of distantly related species genomes.

With near completion of the peach physical map [29] and the development of EST-derived genetic markers, the numbers of sequences that are anchored to *Prunus *genetic maps or the peach physical map have doubled since our previous synteny analysis between *Prunus *and *Arabidopsis*. Also available to us are four homologous *Prunus *BAC sequences and four additional peach BAC sequences. Using these data, we analyzed the degree and the pattern of the conserved syntenic region between *Prunus *and *Populus *or *Arabidopsis*. We also compared the *Prunus *BAC sequences with the partially sequenced *Medicago *genome to gain further insight on the genome evolution of the related plant species.

## Results

### Prunus BAC sequences show extensive conserved synteny with the Populus and Medicago genome, but not with the Arabidopsis genome

Sequences from four *Prunus *BACs, and four additional peach BACs, 028F08, 082I18, pPn31C7, and PpN089G02, were used to assess the degree of conserved synteny with the *Populus*, *Medicago*, and *Arabidopsis *genomes. The four *Prunus *BACs were selected using the same probe to study disease-resistant genes in *Prunus *species, so they potentially represent homologous genomic regions. These include one peach BAC with two contigs (058P54-C23 and 058P54-C24), one apricot BAC (AprC27), and two plum BACs (Plum045O02 and Plum080O24). Gene prediction was conducted with FGENESH [30]. The two peach BACs, 028F08 and 082I18, overlap by 2 kb and two genes, predicted by FGENESH program (see Methods). The combined sequences of the two BACs are 77.7 kb long and contain a total of 38 predicted genes (028F08-082I18_1 to 028F08-082I18_38). The peach BACs pPn31C7 and PpN089G02 were 48.8 kb and 132.2 kb long and had nine (pPn31C7_1 to pPn31C7_9) and 42 (PpN089G02_1 to PpN089G02_42) predicted genes, respectively. The two contigs of a peach BAC and the three *Prunus *BACs that contain putative disease-resistant genes, 058P54-C23, 058P54-C24, AprC27, Plum045O02 and Plum080O24, had 19 (058P54-C23_1 to058P54-C23_19), 9 (058P54-C24_1 to 058P54-C24_9), 11 (AprC27_1 to AprC27_11), 13 (045O02_1 to 045O02_13) and 4 (080O24_1 to 080O24_4) predicted genes, respectively. The sizes of the peach, apricot and plum BAC sequences were 90 kb, 41 kb and 70.1 kb, respectively. Cumulatively, the total length of peach BACs used in our analysis was 350 kb.

The predicted gene sequences in these *Prunus *BACs and their *Populus*, *Medicago *and *Arabidopsis *homologs were used to find conserved syntenic regions. Syntenic groups were identified when the distance between the two adjacent matches was less than 200 kb and when the syntenic regions contain at least four gene pairs. We first detected collinear syntenic regions using the DAGchainer program [31] and then merged the overlapping syntenic regions. This method gave us a more comprehensive view of the syntenic regions which include sections where gene contents are conserved but not the gene order due to small-scale genome rearrangements.

**Figure 1:**
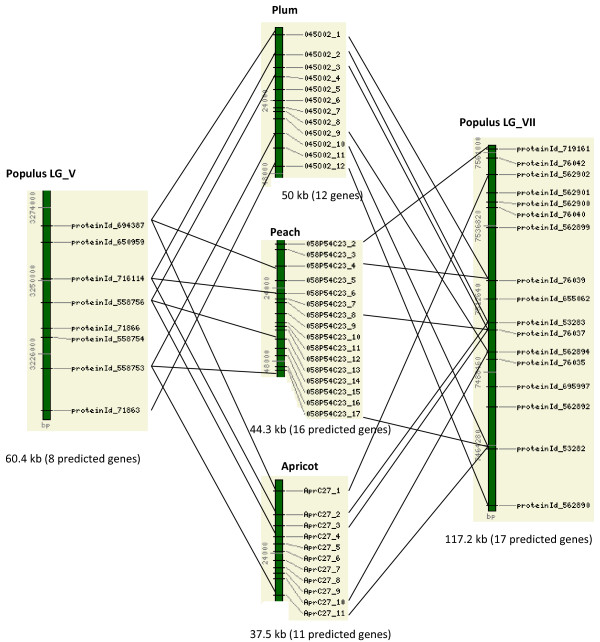
**Conserved synteny between the *Prunus *BACs that contain disease-resistant genes and the genome of *Populus***. All the intervening genes in the syntenic regions are also shown. The numbers on the left side of the bar stand for base pair positions in the *Prunus *BACs or the *Populus *linkage groups. The length of the syntenic region and the total number of predicted genes in the regions are given below the bar.

**Figure 2:**
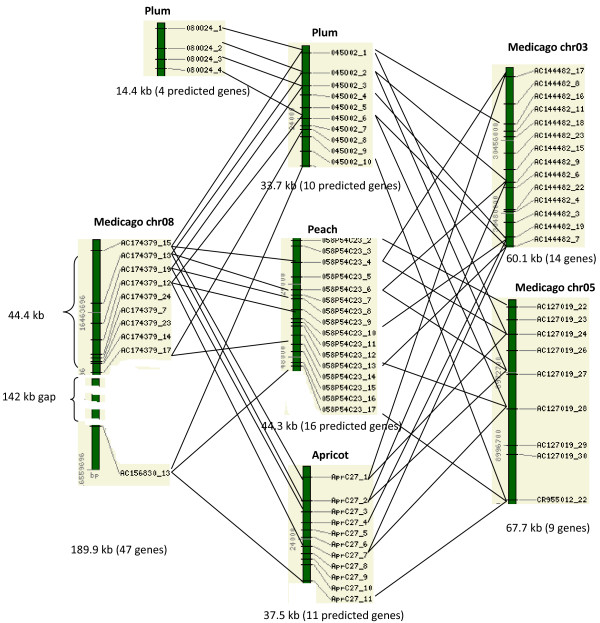
**Conserved synteny between the *Prunus *BACs that contain disease-resistant genes and the genome *Medicago***. All the intervening genes in the syntenic regions, except those in the 142 kb gap, are also shown. The numbers on the left stand for base pair positions in the *Prunus *BACs or the *Medicago *linkage groups. The lengths of the syntenic regions and the total numbers of predicted genes in the regions are given below the bar.

The *Prunus *BACs had syntenic regions in the *Populus *and *Medicago *genome, but no detectable syntenic regions in *Arabidopsis *(Figure 1, 2). Interestingly, two distinct *Populus *genomic regions were detected for each of the *Prunus *BAC regions, and the two corresponded to the duplicated *Populus *genomic regions that were generated by the most recent whole-genome duplication event, which occurred 60–65 million years ago [26]. Figure 1 shows these syntenic regions in LG_V and LG_VII of the *Populus *genome that are conserved with BACs from across the *Prunus *species: peach, plum, and apricot. The syntenic regions span 50 kb in the plum BAC, 44.3 kb in the peach BAC, 37.5 kb in the apricot BAC, and 60.4 kb and 117.2 kb in LG_V and LG_VII of the *Populus *genome, respectively. These three *Prunus *BACs also showed conserved synteny with *Medicago*, in three different chromosomal regions (Figure 2). Detection of more than one syntenic region in *Medicago *correlates with the whole genome duplication event in *Medicago*, which was proposed to have occurred after the split between the legumes and Salicaceae (poplar) and before the separation of *Medicago *and *Lotus *[11]. Cannon et al. [11] shows extensive synteny between chr5 and chr8 of *Medicago*, and we detected two regions in chr5 and chr8 of *Medicago *that are syntenic to the Prunus BACs (Figure 2). The gene order was well conserved in all these syntenic regions without any signs of translocation, but there was a 142 kb gap in the syntenic region in chr08 of the *Medicago *genome (Figure 2). No syntenic regions in *Arabidopsis *were detected for these four *Prunus *BACs.

**Figure 3:**
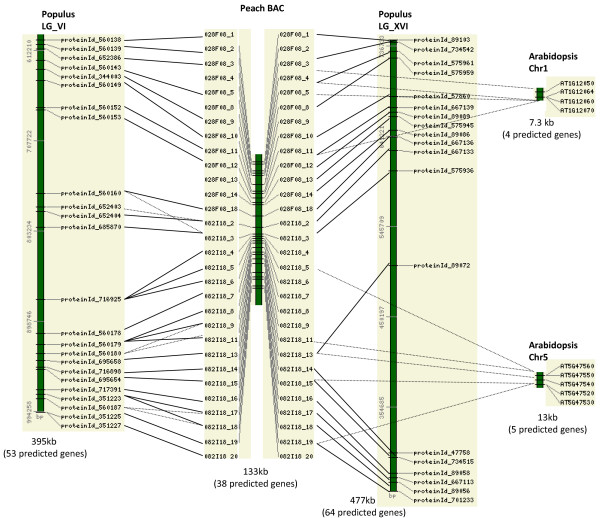
**Conserved syntenic regions between the two overlapping peach BACs and the *Poplulus *or *Arabidopsis *genomes**. When peach sequences match more than one genes in *Populus*, the matches with lower E value (lower PID when the E value is the same) are depicted with dotted lines. The matches between the peach sequences and the *Arabidopsis *genes are depicted with dotted lines. Only the predicted genes showing homology with genes in other genomes, not the intervening genes, are depicted. The numbers on the left side of the bar stand for base pair positions in the *Prunus *BACs, the *Populus *linkage groups, or the *Arabidopsis genome*. The lengths of the syntenic regions and the total numbers of predicted genes in the regions are given below the bar.

**Figure 4:**
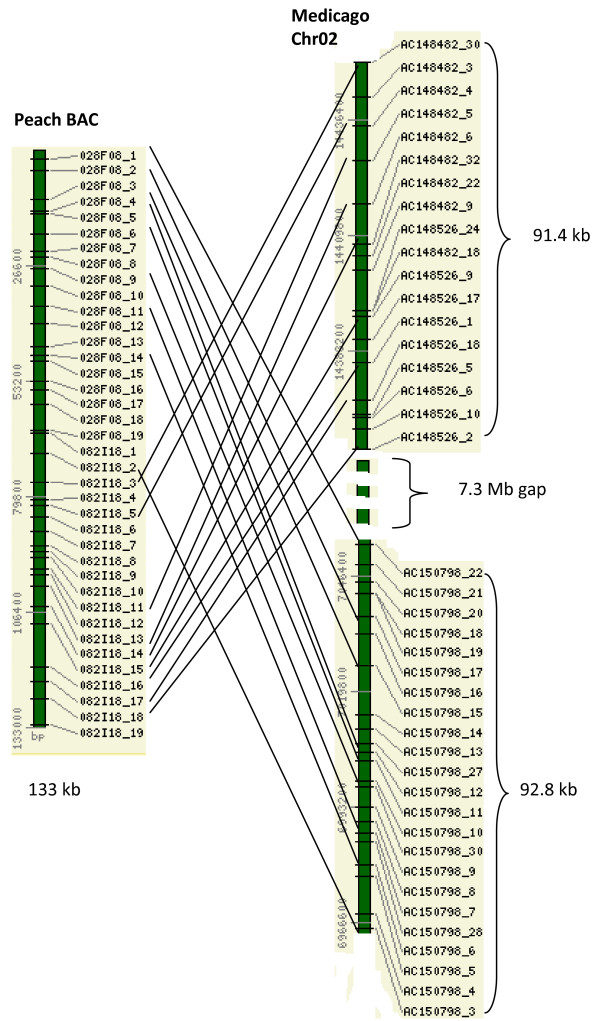
**Conserved syntenic regions between the two overlapping peach BACs and the *Medicago *genome**. All the intervening genes in the syntenic regions, except those in the 7.3 Mb gap, are also shown. The numbers on the left side of the bar stand for base pair positions in the peach BAC or the *Medicago *linkage groups. The lengths of the syntenic regions are given.

The two overlapping peach BACs, 028F08 and 082I18, with a total of 38 predicted genes had remarkably well-conserved syntenic regions in LG_VI and LG_XVI of the *Populus *genome (Figure 3). Of the 38 predicted genes in the combined peach BACs, 26 and 19 showed conserved synteny with the regions in LG_VI and LG_XVI, respectively. In combination, 30 out of 38 predicted peach genes belong to the syntenic groups that are conserved in LG_VI or LG_XVI of the *Populus *genome. The gene order, as well as the gene content, was conserved between *Populus *and peach. The syntenic regions span 133 kb in the peach BACs, and 395 kb in LG_VI and 477 kb in LG_XVI of the *Populus *genome. The actual sizes of the syntenic regions can be larger since the synteny extends to the end(s) of the BAC clone (Figure 3). There were signs of tandem gene duplication in both the *Populus *region and the *Prunus *region (Figure 3). In comparison, two small syntenic regions with only four or five genes spanning 49 kb or 27 kb of the peach BACs and 13 kb or 7.3 kb of the *Arabidopsis *genome were detected in *Arabidopsis *(Figure 3). The overlapping peach BACs also detected a syntenic region in the *Medicago *genome (Figure 4). Two parts in the combined peach BAC detected different regions in the chr02 of *Medicago*, separated by 7.3 Mb, suggesting a translocation event or potential miss-assembly due to the incompleteness of the *Medicago *genome assembly (Figure 4).

**Figure 5:**
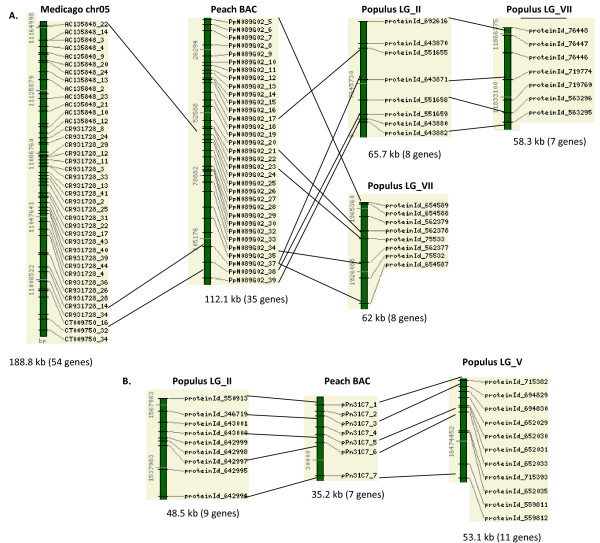
**Conserved syntenic regions between peach BACs and the *Populus *or *Medicago *genome**. These BACs did not have any conserved syntenic regions in the *Arabidopsis *genome. All the intervening genes in the syntenic regions are also shown. The numbers on the left side of the bar stand for base pair positions in the *Prunus *BACs, *Populus *or *Medicago *linkage groups. The lengths of the syntenic regions and the total numbers of predicted genes in the regions are given below the bar.*(A) *Syntenic regions between the peach BAC PpN089G02 and the *Populus *or *Medicago *genome. *(B) *Syntenic regions between the peach BAC pPn31C7 and the *Populus *genome.

The peach BAC PpN089G02 detected three conserved syntenic regions in *Populus *LG_II and LG_VII (Figure 5A). The block in LG_II and a block in LG_VII shared four gene matches to the BAC PpN089G02, and the other region in LG_VII were syntenic to an overlapping region (Figure 5A), suggesting all these three blocks represent a duplicated region that went through selective gene loss. The syntenic regions span 112 kb in the peach BAC PpN089G02, and around 60 kb in all three *Populus *regions. The peach BAC pPn31C7 showed conserved syntenic regions in LG_II and LG_V of the *Populus *genome, which share five gene pairs (Figure 5B). The syntenic regions span 35 kb in the peach BAC pPn31C7, and 48 kb and 53 kb of LG_II and LG_V of the *Populus *genome. In the analysis with the partially sequenced *Medicago *genome, we found one syntenic region that shares four genes with the peach BAC PpN089G02 (Figure 5A). No syntenic regions for the two peach BACs, PpN089G02 and pPn31C7, were detected in the *Arabidopsis *genome.

The fact that we detected microsynteny between all the *Prunus *BACs and *Populus *genome is noteworthy. Also the level of conserved synteny within the individual blocks (synteny quality) between the *Prunus *BACs and *Populus *was strikingly high considering the divergence between these two genomes 99 million years ago [32]. Cannon et al [11] has reported that the quality of all predicted orthologous blocks between the legumes *Medicago truncatula *and *Lotus japonicus *that diverged 40 million years ago, is 54% ± 14%. They calculated "synteny quality" as twice the number of matches divided by the total number of genes in both segments after excluding transposable elements and collapsing tandem duplications. The syntenic quality between *Prunus *BACs and the *Populus *genome ranged from 20 to 44%, without considering the orthology of the blocks or collapsing tandem duplications (Table 1). We only used the syntenic regions where the synteny did not extend to the border of the BAC to calculate the syntenic quality. The syntenic quality between the *Prunus *BACs and *Medicago *genome ranged from 11 to 40% (Table 1).

**Table 1:** Synteny quality within the individual homologus block between the Prunus BACs and Populus or Medicago.

**Species**	**BAC Name**	**Chr**	**No. matches**	**No. genes in BAC**	**No. genes in Chr**	*synteny quality (%)
***Populus***	058P54C23	LG_V	4	11	7	44
		LG_VII	4	16	16	25
	PpN089G02	LG_II	5	34	8	24
		LG_VII	4	34	7	20
		LG_VII	5	33	8	24
***Medicago***	058P54C23	chr3	4	10	14	33
		chr5	5	16	9	40
		chr8	6	14	47	20
	PpN089G02	chr5	4	17	54	11

Only the blocks that the synteny does not extend to the border of the BAC were used.

* Twice the number of matches divided by the total number of genes in both segments

The E values of most of gene matches in the syntenic regions were considerably lower than our cut-off value, 1E-6 (see Methods). 96.3% and 97.1% of the gene matches in *Prunus*-*Populus *and *Prunus*-*Medicago*, respectively, had an E value of less than 1E-10. The median E values were 1E-93 and 1E-109 in *Prunus*-*Populus *and *Prunus*-*Medicago*, respectively.

### Synteny Analysis between map anchored peach sequences and the Populus or the Arabidopsis genome

To assess the degree of macrosynteny conservation, we used two sets of map-anchored *Prunus *sequences in the analysis. One set comprised of 1093 sequences that are anchored to the TxE *Prunus *reference map [33]. The majority of these sequences are peach fruit ESTs but also include sequences from markers that were directly used in mapping. Some ESTs are anchored to multiple positions and the number of anchored sequences in each linkage group is as follows: 281 in G1, 364 in G2, 268 in G3, 424 in G4, 221 in G5, 209 in G6, 65 in G7 and 310 in G8. Another set comprised of peach EST sequences that are anchored to the peach physical map. This data comprised of 2140 EST sequences that are anchored to 1500 BAC contigs and their *Populus *and *Arabidopsis *homologs.

These map-anchored sequences and their *Populus *and *Arabidopsis *homologs were used to find conserved syntenic regions. The syntenic groups were selected when the distance between the two adjacent matches were less than the maximum distance and when the syntenic regions contain at least four gene pairs (See Methods).

There were 8 and 17 syntenic regions containing four or more gene pairs between *Prunus *TxE map and *Populus *or *Arabidopsis*. Between the peach physical map and *Populus *or *Arabidopsis*, 10 and 17 syntenic regions were detected, respectively. Some syntenic groups between *Prunus *and *Arabidopsis *were not collinear, but all the groups between the *Prunus *and *Populus *were collinear (Table 2). In the synteny analysis with the TxE genetic map anchored sequences, the number of gene pairs were similar: four to five in *Populus *and four to seven in *Arabidopsis *genome (Table 2). The syntenic groups span 5 to 15.7 cM in both cases, but span longer in *Populus *(424 kb – 3.26 Mb) than in *Arabidopsis *(197 kb – 1 Mb), reflecting the larger intergenic space in *Populus *than in *Arabidopsis *(see Methods). Similarly, the peach physical map anchored sequences detected longer syntenic regions in *Populus *(158 kb – 1.1 Mb) than in *Arabidopsis *(42 – 812 kb), even though the length of the syntenic regions in the physical map was similar in both cases (Table 2). All ten syntenic groups between the peach and *Populus *had four gene pairs, but eight out of 17 syntenic groups between the peach and *Arabidopsis *had more than four gene pairs (Table 2). As seen in the analysis with the *Prunus *BACs, the E values of most of the gene matches in the syntenic regions were considerably lower than our cut-off value, 1E-6 (see Methods). The median E values were between 2E-39 and 3E-51 (Table 2), and 90% to 100%, depending on the data sets, of the gene matches had the E value of less than 1E-10.

**Table 2:** Characteristics of syntenic blocks conserved between Prunus map anchored sequences and Arabidopsis or Populus.

	**# of syntenic regions**		**# collinear groups**	**# gene pairs**	**Length^1^** **(*Populus *or *Arabidopsis*)**	**Length^1^** **(*Prunus*)**	**Median** **E value**
						
	**before the merge**	**after the merge**					
***Prunus-genetic/Populus (gP)***	8	8	8	4–5	424 kb – 3.26 Mb	5.1 – 15.7 cM^2^ (2.8 – 8.6 Mb)	5.00E-48
***Prunus-genetic/Arabidopsis (gA)***	18	17	16	4–7	197 kb – 1 Mb	4.6 – 15.7 cM^2^ (2.5 – 8.6 Mb)	1.00E-44
***peach-physical/Populus (pP)***	10	10	10	4	158 kb – 1.1 Mb	76–346^3^ (95 – 432.5 kb)	3.00E-51
***peach-physical/Arabidopsis (pA)***	20	17	14	4–9	42–812 kb	65–427^3^ (81.3 – 533.8 kb)	2.00E-39

^1^Range of the lengths of the syntenic blocks.

^2^Numbers in parenthesis are the approximate lengths in peach genome, calculated from the TxE map size 524 cM [39] and the peach genome size 290 Mb[43].

^3^One unit length of the BAC contig corresponds to about 1.25 kb (See Methods).

**Figure 6:**
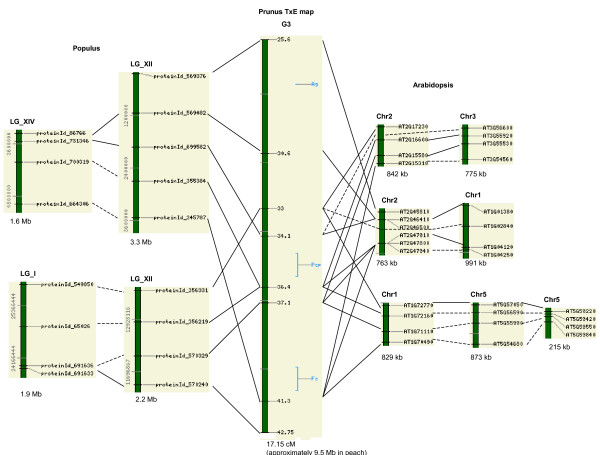
**Syntenic groups covering the three trait loci anchored to G3 of the *Prunus *TxE**. **Map**. The three trait loci shown are Ag (anther color), PcP (polycarpel), and Fc (flower color). There are multiple *Prunus *sequences that are anchored to the same position of the TxE *Prunus *map. The two *Populus *or *Arabidopsis *genes are linked by straight lines when the two genes are matched to the same *Prunus *sequences and by dotted lines when the two genes are matched to different *Prunus *sequences that are anchored to the same position. The approximate length in peach genome is calculated from the TxE map size 524 cM [39] and the peach genome size 290 Mb [43].

Interestingly, despite the smaller number of syntenic groups in *Populus *than in *Arabidopsis*, the syntenic groups in *Populus *actually detected the similar or more distinct regions in the *Prunus *TxE map and physical map, respectively (Table 3). Table 3A shows that the 17 syntenic groups in *Arabidopsis *and the eight syntenic groups in *Populus *were matched to similar regions in three linkage groups of the TxE genetic map, G3, G5 and G8. In other words, the same regions in the TxE map detected more than twice the number of syntenic regions in *Arabidopsis *than in *Populus*, suggestive of multiple genome duplication events in *Arabidopsis*. An example is shown in Figure 6; a region in G3 of the TxE map that displays synteny with four distinct regions in the *Populus *genome and seven distinct regions in the *Arabidopsis *genome. In the analysis with peach physical map anchored sequences, ten groups in *Populus *matched to five different BAC contigs, but 17 groups in *Arabidopsis *matched to only three different BAC contigs (Table 3B). Five and 14 of the syntenic groups were matched to one BAC contig, ctg259 (Table 3B). Even though the BAC contig ctg259 had almost three times more syntenic regions in *Arabidopsis *than in *Populus*, the ctg259 regions that are syntenic to *Arabidopsis *were only 18% larger than those in *Populus *(Table 3B).

**Table 3:** Distribution of syntenic blocks in Prunus TxE map (A) and peach physical map (B).

A							
***Prunus *TxE**	**Synteny with *Populus***				**Synteny with *Arabidopsis***		
	
	***# syntenic groups***		***region in each LG contating syntenic groups (cM)***		***# syntenic groups***	***Region in each LG contating syntenic groups (cM)***	

LG 3	4 (I, XII, XIV)		25.6 – 42.75		7 (Chr1, 2, 3, 5)	25.6 – 41.3	
LG 5	3 (II, XII, XIV)		14 – 19.7		7 (Chr1, 2, 3, 4, 5)	7.4 – 19.7	
LG 8	1 (IX)		26.1 – 35.5		3 (Chr1, 3)	26.1 – 39.1	

***Total***	***8***				***17***		

B							

				**Synteny with *Populus***		**Synteny with *Arabidopsis***	
				
**BAC contig**	**# BACs** **in contig**	contig size*	**# anchored** **sequences**	***# syntenic groups***	***region in each contig contating syntenic groups***	***# syntenic groups***	region in each contig contating syntenic groups*

ctg88	46	117	63			2 (Chr2, Chr3)	17–113
ctg259	381	483	181	5 (II, VI, IX, XIII, XIV)	81–427	14 (Chr1, Chr2, Chr3, Chr4, Chr5)	0–427
ctg468	21	275	6	2 (II, V)	16–201		
ctg524	18	211	11	1 (IV)	59–211		
ctg877	14	218	73			1 (Chr1)	106–129
ctg1008	9	134	7	1 (X)	47–123		
ctg2062	60	254	45	1 (I)	99–211		

***Total***				***10 groups matching to 5 contigs***		***17 groups matching to 3 contigs***	

*One unit length of the BAC contig corresponds to about 1.25 kb (See Methods).

**Figure 7:**
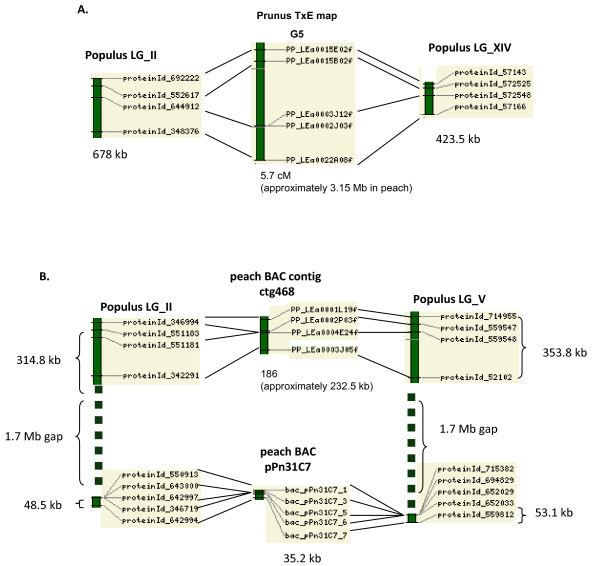
***Prunus *blocks that match to more than one site in the *Populus *genome**. The two conserved syntenic groups are selected when more than three *Prunus *sequences had matches with two different regions in *Populus *or *Arabidopsis*. (A) A region in G5 of the Prunus TxE map matches to regions in LG_II and LG_XIV. (B) A region in peach BAC contig ctg468 matches to regions in LG_II and LG_V. Also shown is a block in peach BAC pPn31C7 that is syntenic to regions in LG_II and LG_V, each region is 1.7 Mb apart from the corresponding region that is syntenic to ctg468. The approximate base pair length in peach genome of the syntenic region in genetic and physical map is calculated from the TxE map size 524 cM [39] and the peach genome size 290 Mb [43], and the total physical length of the peach physical map, 303 Mb, [29] and the total unit length of the BAC contigs, 242772.

We also analyzed for *Prunus *blocks that match to more than one site in the *Populus *or *Arabidopsis *genome. These were detected by selecting syntenic regions that share more than three *Prunus *sequences. We found one *Prunus *block in G5 of the TxE map matching to regions in two different *Populus *linkage groups, LG_II and LG_XIV (Figure 7A). We also found that a *Prunus *block in ctg468 of the physical map matches to regions in LG_II and LG_V (Figure 7B). Tuskan et al [26] have reported that major part of the LG_II is homologous to either LG_V or LG_XIV, generated by the most recent shared whole-genome duplication event. The blocks that we detected in *Prunus *map G5 and peach BAC contig ctg468 showed synteny to the corresponding parts of LG_II that is reported to be homologus to either to LG_V or LG_XIV. Interestingly, the block in the BAC contig ctg468 was syntenic to regions in *Populus *LG_II and LG_V that are each 1.7 Mb distant from the regions that were syntenic to peach BAC pPn31C7 (Figure 5B, Fig 7B). This suggests that BAC contig ctg468 and the peach BAC pPn31C7 may be linked. We did not detect any *Prunus *blocks sharing more than three sequences and matching to two different regions even though we observed many cases of multiple *Arabidopsis *regions matching to similar *Prunus *regions. All the multiple *Arabidopsis *regions that match to similar regions in *Prunus *share none or less than three sequences, suggesting *Arabidopsis *genome underwent severe gene loss after multiple genome duplication.

### Syntenic regions around agronomically important trait loci in the Prunus TxE map

The position of 28 major trait loci affecting agronomic characters found in various *Prunus *species have been previously established in the TxE map using the data from different linkage maps anchored with the TxE reference map [3]. We were interested in finding any syntenic regions conserved between the TxE *Prunus *map and the *Arabidopsis *or *Populus *genomes covering these trait loci. Several syntenic regions matched to trait-loci containing regions in G3 of the *Prunus *TxE map (Figure 6). The trait loci contained in these syntenic regions are Ag for anther color of peach and almond (yellow/anthocyanic), PcP for the polycarpel trait of peach [34], and Fc for flower color (pink/pale pink) in peach [35]. All these syntenic regions span 8.3 cM to 15 cM in the TxE map and contained either two or three trait loci (Figure 6). Among the four *Populus *blocks that matched to these regions, two blocks in LG_XII and LG_XIV seem to represent duplicated blocks, since two genes matched to the same peach sequences and two genes matched to the sequences that are anchored to the same position in the TxE map. The other two *Populus *blocks in LG_I and LG_XII may also represent duplicated regions since one of the genes matched to the same peach sequence and the rest matched to the sequences that are anchored to the same position in the TxE map. There were seven *Arabidopsis *blocks that matched to these regions. Some blocks share one to two genes that match to the same peach sequences and other blocks share two to three genes that match to the sequences that are anchored to the same position in the TxE map. The complex network of synteny in *Arabidopsis *in this region is also indicative of multiple independent genome duplications and rearrangements in the *Arabidopsis *genome.

### Simulation study

To determine whether the syntenic groups we report were detected by chance, we tested the statistical significance for each group. Both *Populus *and *Arabidopsis *genomes were randomized by leaving the locations the same but permuting the gene names. We analyzed 1000 simulated *Populus *and *Arabidopsis *genomes for the occurrence of each conserved syntenic group and calculated the probability of the match occurring by chance. The probability of the association by chance was less than 0.1% for all the syntenic groups between *Arabidopsis *or *Populus *and the *Prunus *physical map, less than 1% for groups between *Populus *and the *Prunus *genetic map, and less than 5% for the groups between *Arabidopsis *and the *Prunus *genetic map. The numbers of syntenic groups at various significance thresholds are shown in Table 4.

**Table 4:** Number of syntenic groups between Populus or Arabidopsis genomes and Prunus genetic map or peach physical map that are detected at various significance thresholds.

	**Significance threshold**			
	
**Syntenic Group**	***> 99.9%***	***> 99%***	***> 95%***	***Total # of groups***
***Prunus-genetic/Populus (gP)***	7	8	8	8
***Prunus-genetic/Arabidopsis (gA)***	8	14	17	17
***peach-physical/Populus (pP)***	10	10	10	10
***peach-physical/Arabidopsis (pA)***	17	17	17	17

## Discussion

We compared the level of conserved synteny between the *Prunus *genome and the whole genome sequences of two model organisms, *Arabidopsis *and *Populus*, and the partially sequenced *Medicago *genome. Since the whole genome sequence of peach or other *Prunus *sequences is not currently available, we employed available *Prunus *BAC sequences to assess the level of conserved microsynteny and map-anchored *Prunus *sequences to assess the pattern of synteny throughout the genome. In the *Populus *genome, we found well-conserved syntenic regions in all the BACs studied, and the syntenic regions cover almost the entire length of the BACs in some cases. All the BACs showed conserved synteny with two regions in two different *Populus *chromosomes, supporting the proposed event of the whole genome duplication in *Populus *[26]. The conserved gene content in the two different *Populus *genomes showed that different sets of genes were lost in the duplicated *Populus *regions. The order of genes in these BACs and both of the *Populus *regions were very well conserved, without signs of genome translocations. The synteny quality within each block was also considerable. On the contrary, we did not detect that level of microsynteny between *Prunus *BACs and the *Arabidopsis *genome. The two microsyntenic regions between these contained only four and five gene matches. We also found well-conserved syntenic regions between *Prunus *BACs and the partially sequenced *Medicago *genome. Interestingly, the homologous BACs of plum, peach, and apricot detected three syntenic regions in three different chromosomes of *Medicago*. These three regions may be indicative of large-scale or segmental duplication events in the evolutionary history of *Medicago*, in addition to the whole genome duplication event proposed to have occurred within the rosid I clade. The other two peach BACs detected only one syntenic region in *Medicago*. This may be explained by the fact that the *Medicago *Genome Release 1.0 constitutes only 38–47% of the entire genome. Another possibility is that these peach BACs do have only one conserved syntenic region in *Medicago *genome. It has shown that the level of internal synteny within both *Medicago *and *Lotus *is much lower than the intergenomic synteny between the two, presumably through significant gene loss and rearrangement after the whole genome duplication event [11].

Our study analyzed a fine-level microsynteny between *Prunus *and the three model dicot organisms. Our results substantiate our previous findings that the level of gene-order conservation between peach and *Arabidopsis *is very fragmentary [17,36]. The microsynteny between the *Prunus *and *Populus *genome, however, is much better conserved, promising the utility of the *Populus *genome in the study of the *Prunus *genome and vice versa when the peach genome is sequenced (underway by the Joint Genome Institute). Our results also suggest that the *Prunus *genome may be closer to *Populus *than to *Medicago*, which is evolutionarily closer to *Prunus *than *Populus*. Similar results have been reported; two melon BACs showed more conserved synteny to *Populus *than to the evolutionarily closer *Medicago *[27]. Lai et al. [28] reported that ordered papaya BAC end sequences showed a higher level of synteny with poplar than with the more closely related *Arabidopsis *which both belong to the Brassicales.

We also employed *Prunus *sequences that are anchored to the *Prunus *genetic map or the peach physical map to study the level of conserved synteny at the whole genome level. Only the completely sequenced genomes of *Populus *and *Arabidopsis *were used in this analysis. A number of syntenic regions that contain several gene pairs were detected between *Prunus *map anchored sequences and both *Populus *and *Arabidopsis *genomes. The number of the syntenic regions was actually higher in *Arabidopsis *than in *Populus*. However, in most cases, multiple *Arabidopsis *regions were syntenic to the overlapping regions in the *Prunus *TxE map or peach BAC contigs, resulting in a complex network of synteny. In contrast, the syntenic regions in *Populus *detected more distinct regions in *Prunus*. We found blocks in both the *Prunus *genetic map and peach physical map that were syntenic to two different homologous regions in *Populus *genome. However, we did not find any *Prunus *regions that detected homologous regions in the *Arabidopsis *genome despite the fact that multiple *Arabidopsis *genomic regions were syntenic to overlapping *Prunus *regions. Our findings are consistent with previous reports that the *Arabidopsis *genome went through multiple large genome duplication events followed by frequent gene loss [37,38]. The multiple *Arabidopsis *genome regions with synteny to overlapping *Prunus *regions, which we observed, may represent the duplicated regions that subsequently went through gene loss. Simillion et al. [37] have reported that high frequency of gene loss after duplication in *Arabidopsis *reduces collinearity and that, at extreme, the duplicated regions no longer share homologous genes. They have shown that they could detect these highly degenerated duplicated blocks by indirect comparison with other segments. The synteny obscured by severe subsequent gene loss can impede the transfer of genomic knowledge from one species to another, suggesting the potential problem of using *Arabidopsis *in the study of other species such as those in *Prunus*. In contrast, the *Populus *genome is reported to be a more stable and homologous genome; blocks that have arisen from the most recent genome-wide duplication event can be clearly detected [26]. Our results corroborate these previous findings and suggest that *Populus *is better model to study synteny with *Prunus *genomes.

## Conclusion

We report the evidence of well-conserved microsynteny between *Prunus *BACs and two plant model species, *Populus *and *Medicago*. The observed network of synteny supported the whole genome duplication events, proposed to have occurred in *Populus *and *Medicago*. Interestingly, the level of synteny conservation seemed higher between *Prunus*-*Populus *than in *Prunus*-*Medicago*, even though *Medicago*, rather than *Populus*, is evolutionarily closer to *Prunus*. In comparison, the level of conserved synteny between the *Prunus *BAC and *Arabidopsis *was insignificant. We also analyzed the extent of conserved synteny between *Prunus *map-anchored sequences and the completely sequenced genomes of *Populus *and *Arabidopsis*. We detected a number of syntenic regions that contain several gene pairs between *Prunus *map anchored sequences and both *Populus *and *Arabidopsis *genomes. In comparison to the synteny between *Populus *and *Prunus*, we observed a more complex network of synteny between *Arabidopsis *and *Prunus *in which multiple *Arabidopsis *regions shows synteny to overlapping regions in *Prunus*.

## Methods

### Data Acquisition and Annotation

Three sets of *Prunus *sequences were used in the analysis of conserved synteny between *Prunus *and the whole genome sequences of *Arabidopsis *and *Populus*: *Prunus *BAC sequences, *Prunus *sequences anchored to the *Prunus *reference map [1,39], and the peach EST sequences anchored to the peach physical map [29]. *Prunus *BAC sequences were also compared with the partially sequenced *Medicago *genome [11].

The sequences of four peach BACs, 028F08 (AC154900), 082I18 (AC154901), pPn31C7 (AF467900), and PpN089G02 (DQ863257), were downloaded from Genbank. The sizes of the sequences were 66.2 kb, 73.3 kb, 48.8 kb, and 132.3 kb, respectively. We also downloaded the sequences of four *Prunus *BACs that contain a putative disease resistant gene from the Genome Database for Rosaceae (GDR) [40]. These include one peach BAC with two contigs (058P54-C23 and 058P54-C24), one apricot BAC (AprC27), and two plum BACs (Plum045O02 and Plum080O24). The two peach BACs, 028F08 (AC154900), 082I18 (AC154901) are a contiguous genomic segment. Gene prediction was done using the FGENESH program [30].

*Prunus *sequences that are anchored to the TxE *Prunus *reference map were downloaded from GDR. The majority of the 1093 anchored sequences were derived from peach fruit ESTs [32]. Some ESTs were directly anchored to the map by co-hybridization to BACs with genetic markers, and others were indirectly anchored through hybridization to BACs that belong to the same BAC contig that contain anchored genetic markers. The positions (cM) of the genetic markers were used as the positions for the genetically anchored ESTs on the map. The set also included sequences of markers that are directly used in mapping. The third set, also downloaded from GDR, comprised of 2140 EST sequences that were anchored to 1500 BAC contigs of the peach physical map. The position of the individual BACs in the BAC contigs were used as the positions ESTs in the peach physical map. For the ESTs that are anchored to multiple overlapping BACs in a BAC contig, the innermost left and right positions were assigned.

The sequence data (TAIR6_pep_20060907) and the chromosome coordinate data (sv_gene.data) of the *Arabidopsis *translated proteins were downloaded from the *Arabidopsis *Information Resources (TAIR) database [41]. The sequence data (Poptr1_FilteredModels_proteins.fa) and the chromosome coordinate data (Poptr1_FilteredModels.gff) of the *Populus *translated proteins were downloaded from the JGI web site . *Medicago *genome release version 1.0 data was downloaded from . International *Medicago *Genome Annotation Group (IMGAG) annotated protein sequences (MTpep1.txt) were used for our analysis.

### Detection of conserved syntenic regions

The predicted protein sequences of the *Prunus *BACs were compared with the *Arabidopsis*, *Populus*, and *Medicago *proteins by pairwise comparison using the BLASTP program. The top ten matches with an E value less than 10^-6 ^were used for further analysis. Syntenic groups were selected when the distance between the two adjacent matches were less than 200 kb and when the syntenic regions contain at least four gene pairs.

Mapped *Prunus *sequences that are homologous to the *Arabidopsis *or *Populus *proteins were determined by pairwise comparison using the BLASTX program. The top ten matches with E value less than 10^-6 ^were used for further analysis. The syntenic groups were selected when the distance between the two adjacent matches were less than the maximum distance and when the syntenic regions contain at least four gene pairs. For the synteny analysis between the TxE genetic map and *Populus *or *Arabidopsis*, the maximum distance was set as the approximate genomic distance that covers about 100 genes – 1 Mb for *Populus *and 0.5 Mb for *Arabidopsis*. This was approximated by the genome size, 550 Mb for *Populus *[26] and 157 Mb for *Arabidopsis *[42], and the predicted number of genes, 45,555 for *Populus *(annotation v1.1) and 32,041 (TAIR7 Genome release) for *Arabidopsis*. For sequences anchored in the TxE *Prunus *genetic map, we selected a less strict parameter, 5 cM, as the maximum distance since most of the sequences are not directly mapped, but are indirectly anchored to the position by the association with other mapped markers (see above). 5 cM is 0.95% of the TxE map (524 cM) [39] and 0.5 Mb that we used for *Arabidopsis *is about 0.35% of the genome (157 Mb). We used a smaller maximum distance – 0.5 Mb for *Populus *and 0.25 Mb for *Arabidopsis *– for the synteny analysis between the peach physical map and *Populus *or *Arabidopsis*, since the sequences are positioned to BAC contigs, which are much smaller than chromosomes or linkage groups. The maximum distance in peach BAC contig was set to 200 unit lengths (unit given in the FPC output), which corresponds to 0.25 Mb. One unit length corresponds approximately to 1.25 kb, estimated from the total physical length of the peach physical map, 303 Mb, [29] and the total unit length of the BAC contigs, 242772.

We employed DAGchainer [31] to detect collinear segments conserved in *Prunus *and other genomes of *Arabidopsis*, *Medicago *and *Populus*. When we used map-anchored *Prunus *sequences, we found many cases in which multiple collinear segments, detected by the software, reside in the same or overlapping genome regions. This occurs when the two genomes in comparison contain syntenic regions in which gene contents are conserved but the gene order is not well conserved, which is caused by differential small genome rearrangements such as conversions, translocations and duplications in the evolutionary history of two genomes. In these regions, DAGchainer can detect more than one collinear syntenic region in the same genomic block by selecting the gene pairs with the conserved order. To solve this problem, we merged the overlapping syntenic regions that are generated by DAGchainer. The syntenic groups were merged when they overlap or when they are separated by less than the maximum distance between the two adjacent matches in both genomes. The merged syntenic groups represent syntenic regions in which the gene contents are conserved but not the gene order. This procedure provides us with a more accurate assessment of the degree of conserved synteny between the two genomes. The *Prunus *blocks that match to more than one site in the *Populus *or *Arabidopsis *genome were detected by selecting syntenic regions that share more than three *Prunus *sequences. All the in-house developed scripts are available to the public when requested.

### Evaluation of the conserved syntenic regions

To determine whether the syntenic groups we report were detected by chance, we tested the statistical significance for each group. Both of the *Populus *and *Arabidopsis *genomes were randomized by leaving the locations the same but permuting the gene names. We analyzed 1000 simulated *Populus *and *Arabidopsis *genomes for the occurrence of each conserved syntenic group and calculated the probability of the match occurring by chance.

## List of abbreviations

GDR: Genome Database of Rosaceae; TAIR: The *Arabidopsis *Information Resources; IMGAG: International *Medicago *Genome Annotation Group.

## Authors' contributions

SJ designed the protocol for synteny analysis and the statistical analysis, designed and developed scripts, performed the research, analyzed the data and wrote the paper. DJ originally sequenced four *Prunus *BACs that contain a putative disease resistant gene and participated in the design of the study. IC and TL wrote the scripts for parsing the DAGchainer outputs to further restrict the gap size between the genes in a syntenic region, to merge overlapping syntenic regions, to name the syntenic regions, and to report the results. AA conceived of the study and critically revised the manuscript. BS participated in the design of the study and critically revised the manuscript. DM conceived of the study and participated in its design and coordination, and critically revised the manuscript.
